# Functional polymorphisms in *pre-miR146a* and *pre-miR499* are associated with systemic lupus erythematosus but not with rheumatoid arthritis or Graves’ disease in Mexican patients

**DOI:** 10.18632/oncotarget.19621

**Published:** 2017-07-27

**Authors:** Isidro Alemán-Ávila, Mayra Jiménez-Morales, Olga Beltrán-Ramírez, Rosa Elda Barbosa-Cobos, Silvia Jiménez-Morales, Fausto Sánchez-Muñoz, Guillermo Valencia-Pacheco, Luis M. Amezcua-Guerra, Yaneli Juárez-Vicuña, Dulce Milagro Razo-Blanco Hernández, María Concepción Aguilera-Cartas, Ricardo F. López-Villanueva, Oscar Peralta-Zaragoza, Carlos Tovilla-Zárate, Julian Ramírez-Bello

**Affiliations:** ^1^ Endocrine and Metabolic Disease Unit Research, Hospital Juarez of Mexico, Mexico City, Mexico; ^2^ Superior School of Medicine Postgraduate Program, National Polytechnic Institute, Mexico City, Mexico; ^3^ Rheumatology Department, Hospital Juarez of Mexico, Mexico City, Mexico; ^4^ Oncology Department, National Institute of Genomic Medicine, Mexico City, Mexico; ^5^ Immunology Department, National Institute of Cardiology, Mexico City, Mexico; ^6^ Hematology Laboratory, Regional Research Center, Autonomous University of Yucatan, Yucatan, Mexico; ^7^ Research Direction, Hospital Juarez of Mexico, Mexico City, Mexico; ^8^ Endocrine Department, Hospital Juarez of Mexico, Mexico City, Mexico; ^9^ Rheumatology Department, Regional Hospital General (ISSSTE), Health Service Yucatan, Yucatan, Mexico; ^10^ Direction of Chronic Infections and Cancer, Research Center in Infection Diseases, National Institute of Public Health, Cuernavaca, México; ^11^ Multidisciplinary Academic Division of Comalcalco, Juarez Autonomous University of Tabasco, Comalcalco, Mexico

**Keywords:** systemic lupus erythematosus, rheumatoid arthritis, Graves’ disease, microRNA gene, susceptibility

## Abstract

Recently, different microRNA (miRNA) gene polymorphisms have been evaluated in patients with rheumatoid arthritis (RA), systemic lupus erythematosus (SLE), and Graves’ disease (GD). In the present study, we examined three single-nucleotide polymorphisms (SNPs) located in the *pre-miR-146a* (rs2910164G/C), *pre-miR-196a-2* (rs11614913C/T), and *pre-miR-499* (rs3746444A/G) genes. Our study population included 900 Mexican patients with RA, SLE, or GD, as well as 486 healthy control individuals with no family history of inflammatory or autoimmune diseases. Genotyping was performed using TaqMan probes and a 5′ exonuclease assay. None of the investigated SNPs were associated with RA or GD susceptibility under any genetic model (co-dominant, recessive, or dominant). Genotype and allele frequencies of the *miR-196a-2* rs11614913C/T polymorphism were similar between SLE cases and controls. In contrast, the *miR-146a* rs2910164G/C and *miR-499* rs3746444A/G polymorphisms were associated with SLE susceptibility. These SNPs were not associated with lupus nephritis (LN). Our results suggest that polymorphisms in *miR-146a, miR-196a-2*, and *miR-499* are not associated with RA or GD susceptibility. This is the first report documenting that the *miR-146a* rs2910164G/C and *miR-499* rs3746444 polymorphisms are associated with SLE susceptibility but not with LN.

## INTRODUCTION

Autoimmune diseases (AIDs) constitute a heterogeneous group of pathologies characterized by loss of immunological tolerance, production of autoantibodies, and increased expression of cytokines with inflammatory activity [1]. In the United States, AIDs affect about 5–8% of the general population, with most showing a higher prevalence in women than in men [2]. Rheumatoid arthritis (RA) is a chronic inflammatory autoimmune disease characterized by synovial inflammation, which leads to joint tissue destruction and functional disability [3]. Systemic lupus erythematosus (SLE) is the prototype of a multi-systemic AID, and is characterized by loss of immunological tolerance against self-antigens and production of pathogenic autoantibodies, ultimately resulting in damage to multiple organ systems [4]. Graves’ diseases (GD) is an organ-specific AID, in which the major antigenic target is the thyroid-stimulating hormone receptor (TSHR). TSHR-autoantibodies bind to TSHR, mimicking the action of its ligand (TSH) and causing hyperthyroidism [5].

Although the etiologies of RA, SLE, and GD remain unclear, the identified risk factors include gender; genetic background; and environmental agents, such as geography, climate, endemic microbes, and socio-cultural practices, including smoking, lifestyle, and dietary habits [6–7]. Investigations using a candidate gene approach and genome-wide association studies have demonstrated that RA, SLE, and GD susceptibility is conferred by different variants of protein-coding genes, including *HLA-class II, PTPN22, TNFAIP3, STAT4*, *TNFRSF14, BLK*, and *TRAF1* [8–10]. Genetic risk factors for these AIDs may also include single-nucleotide polymorphisms (SNPs) in non-coding RNA genes, including the microRNAs (miRNAs) *146a, miR-196a-2* and *miR-499* [11–17].

RNA polymerase II initially transcribes miRNA genes, producing primary miRNAs (pri-miRNAs) of approximately 2 kb in length. In the nucleus, these pri-miRNAs are processed by RNase III Drosha and the DiGeorge syndrome chromosomal region 8, Microprocessor Complex Subunit (DGCR8; DiGeorge syndrome chromosomal region 8) protein, generating a precursor miRNA (pre-miRNA) of approximately 100 bp. These pre-miRNAs are transported from the nucleus to the cytoplasm via exportin-5. In the cytoplasm, pre-miRNAs are cleaved by the RNase III enzyme Dicer, in complex with the human immunodeficiency virus transactivating response RNA-binding protein (TRBP), ultimately forming small non-coding RNAs of approximately 19–22 nucleotides, termed mature miRNAs [18–22]. Mature miRNAs function in the negative regulation of gene expression, acting at the post-transcriptional level by binding to the 3′ untranslated region (3′UTR) of target messenger RNAs (mRNAs), thus preventing their translation into proteins [22–25].

Various miRNAs play important roles in regulating a wide array of biological functions, including proliferation, apoptosis, differentiation, immune response, and inflammation [22–27]. With regards to AIDs, miR-146a is abnormally expressed in RA-affected tissues, including synovial fibroblasts, synovial tissue, serum, and peripheral blood mononuclear cells (PBMCs) [28–33]. This miRNA also shows abnormal expression in PBMCs and monocytes from SLE patients [33, 34], and in PBMCs from patients with Graves’ ophthalmopathy (GO). Moreover, patients with active GO show lower serum miR-146a levels than those with inactive GO [35, 36]. Both miR-196a and miR-499 are thought to be involved in autoimmune and inflammatory diseases, because they promote the expressions of several relevant proteins, including interleukin (IL)-23a, IL-6, IL-2, C-reactive protein, IL-18R, and IL-2R. Thus, both miRNAs could potentially contribute to the pathogenesis and disease progression of several AIDs [37–39].

Functional SNPs located in miRNA genes (miR-SNPs) could potentially affect pri-miRNA transcription or pri-miRNA/pre-miRNA processing, or could disrupt miRNA–mRNA interactions if located in the mature miRNA sequence or in miRNA binding sites [40–42]. The functional miR-SNP rs2910164G/C, located in *pre-miR-146a*, has been evaluated in RA patients from different populations, yielding controversial results. Yang et al. [11], Hashemi et al. [12], and El-Shal et al. [13] reported that this polymorphism was not associated with RA susceptibility; however, Zhou et al. [43] found that the GG genotype was significantly associated with RA among women. This SNP has not been associated with SLE or GD susceptibility in patients from different populations [16, 44, 45]. Another functional miR-SNP, *miR-499* rs3746444A/G, is reportedly associated with RA susceptibility, severity, and disease activity in Iranian and Egyptian populations, but not in a Chinese population [11–13, 46]. This polymorphism also showed no association with SLE susceptibility in a Chinese population [44]; however, it was recently found to be associated with GD susceptibility in a Chinese population [16]. The functional *miR-196a-2* rs11614913C/T SNP has only been evaluated in Egyptian patients with RA, and no evidence of association was detected [46].

In the present case-control study, we evaluated whether the polymorphisms *pre-miR-146a* rs2910164G/C, *pre-miR-196a-2* rs11614913C/T, and *pre-miR-499* rs3746444A/G conferred risk for RA, SLE, and GD in a sample of Mexican patients. We also evaluated whether these polymorphisms were associated with lupus nephritis (LN).

## RESULTS

### Demographic features of cases and controls

This study included 900 patients with AIDs: 412 with RA (378 female, 34 male), 407 with SLE (384 female, 23 male), and 81 with GD (72 female, 9 male). The study also included 486 healthy control individuals (431 female, 55 male). Table 1 presents demographic features of the RA, SLE, and GD patients, and the controls.

**Table 1:** Demographic characteristics of the patients with RA, SLE and GD from Mexico included in this study

	Controls (n=486)	RA (n=412)	SLE (n=407)	GD (n=81)
Age (years)	50.9	51.8	39.9	36.2
**SD** ^*^	(±7.3)	(±13.6)	(±12.6)	(±10.8)
Gender:				
Female n (%)	431 (88.7)	378 (91.7)	384 (94.3)	72 (88.9)
Male n (%)	55 (11.3)	34 (8.3)	23 (5.7)	9 (11.1)

^*^ SD: standard deviation;

RA: rheumatoid arthritis; SLE: systemic lupus erythematosus; GD: Graves’ disease.

### Hardy-Weinberg equilibrium (HWE) in the study population

In this case-control study, the genotype frequencies of the *miR-146a* rs2910164G/C, *miR-196a-2* rs11614913C/T, and *miR-499* rs3746444A/G polymorphisms were in HWE (Tables 2–4).

**Table 2:** Genotype and allelic frequencies of the *miR-146a rs2910164G/C* polymorphism and association analysis in RA, SLE and GD patients

SNP ID	Population	Allele	Genotype n (%)						Allele n (%)				
		12	11	12	22	OR (11vs22)	95% CI	*p*	1	2	OR (1vs2)	95% CI	*p*
*miRNA146a*	Controls	G C	218(44.9)	222(45.7)	46(9.4)	—	—	—	658(67.7)	314(32.3)	—	—	—
(rs2910164)	RA^*^		168(41.0)	196(47.8)	46(11.2)	1.3	(0.82-2.04)	0.261	532(64.9)	288(35.1)	1.1	(0.93-1.38)	0.208
	SLE		168(41.3)	179(44.0)	60(14.7)	1.7	(1.09-2.61)	**0.017**	515(69.2)	229(30.8)	1.2	(1.00-1.48)	**0.049**
	GD		29(35.8)	44(54.3)	8(9.9)	1.3	(0.56-3.04)	0.53	102(63.0)	60(37.0)	1.2	(0.87-1.74)	0.235

OR: odds ratio; CI: confidence interval; p: p value; RA: rheumatoid arthritis; SLE: systemic lupus erythematosus; GD: Graves’ disease.

Significant *p*-values are reported in bold type.

H-WE *p* values to controls (*p*=0.33), RA (*p*=0.32), SLE (*p*=0.28), and GD (*p*=0.14).

^*^ Two RA samples were not genotyped.

**Table 3:** Genotype and allelic frequencies of the *miR-196a-2 rs11614913C/T* polymorphism and association analysis in RA, SLE, GD patients and controls

SNP ID	Population	Allele	Genotype n (%)						Allele n (%)				
		12	11	12	22	OR (11vs22)	95% CI	*p*	1	2	OR (1vs2)	95% CI	*p*
*miRNA196a2*	Controls	C T	182(37.5)	230(47.3)	74(15.2)	—	—	—	594(61.1)	378(38.9)	—	—	—
(rs11614913)	RA		142(34.5)	213(51.7)	57(13.8)	1.0	(0.66-1.49)	0.951	497(60.3)	327(39.7)	1.0	(0.86-1.25)	0.731
	SLE^*^		155(38.3)	189(46.7)	61(15.0)	1.0	(0.65-1.44)	0.873	499(61.6)	311(38.4)	1.0	(0.81-1.19)	0.831
	GD^**^		28(35.0)	39(48.8)	13(16.2)	1.1	(0.56-2.32)	0.714	95(59.4)	65(40.6)	1.1	(0.76-1.51)	0.676

OR: odds ratio; CI: confidence interval; p: p value; RA: rheumatoid arthritis; SLE: systemic lupus erythematosus; GD: Graves’ disease.

H-WE *p* values to controls (*p*=0.93), RA (*p*=0.10), SLE (*p*=0.79), and GD (*p*=0.93).

^*^ Two SLE samples were not genotyped. ^*^ One GD sample was not genotyped.

**Table 4:** Genotype and allelic frequencies of the *miR-499 rs3746444A/G* polymorphism and association analysis in RA, SLE, GD patients and controls

SNP ID	Population	Allele	Genotype n (%)						Allele n (%)				
		12	11	12	22	OR (11vs22)	95% CI	*p*	1	2	OR (1vs2)	95% CI	*p*
*miRNA499*	Controls	A G	425(87.5)	60(12.3)	1(0.2)	--	--	--	910(93.6)	62(6.4)	--	--	--
(rs3746444)	RA		352(85.4)	57(13.8)	3(0.8)	1.2	(0.77-1.69)	0.489	761(92.4)	63(7.6)	1.2	(0.84-1.74)	0.293
	SLE		336(82.6)	71(17.4)	0(0%)	1.5	(1.03-2.17)	**0.033**	743(91.3)	71(8.7)	1.4	(0.98-2.00)	0.060
	GD		69(85.2)	12(14.8)	0(0%)	1.2	(0.63-2.40)	0.541	150(92.6)	12(7.4)	1.2	(0.62-2.23)	0.623

OR: odds ratio; CI: confidence interval; p: p value; RA: rheumatoid arthritis; SLE: systemic lupus erythematosus; GD: Graves’ disease.

Significant P-values are reported in bold type.

H-WE *p* values to controls (*p*=0.46), RA (*p*=0.68), SLE (*p*=0.054), and GD (*p*=0.47).

### Case-control genetic association analysis

The statistical power values were 93.4% for RA, 93.3% for SLE, and 34.3% for GD. The genotype and allele frequencies of the *miR-146a* rs2910164G/C, *miR-196a-2* rs11614913C/T, and *miR-499* rs3746444A/G polymorphisms were similar between RA and GD patients and controls. Case-control analysis revealed no association between these three SNPs and RA or GD susceptibility Tables 2, 4, and 6, even when using recessive and dominant genetic models (Tables 2–7). Genotype and allele frequencies of the *miR-196a-2* rs11614913C/T SNP were also similar between SLE patients and controls. However, the *miR-146a* rs2910164G/C and *miR-499* rs3746444A/G polymorphisms were associated with SLE susceptibility (Tables 2 and 4). Comparison of the *miR-146a* rs2910164G/C and *miR-499* rs3746444A/G polymorphisms between SLE patients and controls revealed associations with SLE susceptibility under both the recessive and dominant genetic models (Tables 5 and 7). None of the investigated SNPs showed association with LN (Table 8). Analysis with gender stratification showed no association between the three analyzed polymorphisms and RA, SLE, or GD (data not shown).

**Table 5:** Association analysis between the *miR-146a rs2910164G/C* polymorphism in RA, SLE and GD patients under different genetic models

Recessive model								
SNP ID	Genotypes	Controls	RA	OR, 95 % CI, *p*	SLE	OR, 95 % CI, *p*	GD	OR, 95 % CI, *p*
***miR-146ª (rs2910164) G/C***	GG + GC	440 (90.5)	364 (88.8)	—	347 (85.3)	—	73 (90.1)	—
	CC	46 (9.5)	46 (11.2)	1.2	60 (14.7)	1.7	8 (9.9)	1.0
				(0.78-1.86)		(1.10-2.49)		(0.48-2.31)
				0.389		**0.015**		0.907
	**Dominant model**							
	**Genotypes**	**Controls**	**RA**	**OR, 95 % CI, *p***	**SLE**	**OR, 95 % CI, *p***	**GD**	**OR, 95 % CI, *p***
	GG	218 (44.9)	168 (41.0)	—	168 (41.3)	—	29 (35.8)	—
	GC + CC	268 (55.1)	242 (59.0)	1.2	239 (58.7)	1.2	52 (64.2)	1.5
				(0.90-1.53)		(0.89-1.51)		(0.84-2.38)
				0.243		0.282		0.128

OR: odds ratio; CI: confidence interval; p: p value; RA: rheumatoid arthritis; SLE: systemic lupus erythematosus; GD: Graves’ disease.

Significant P-values are reported in bold type.

**Table 6:** Association analysis between the *miR-196a-2 rs11614913C/T* polymorphism in RA, SLE and GD patients under different genetic models

Recessive model								
SNP ID	Genotypes	Controls	RA	OR, 95 % CI, *p*	SLE	OR, 95 % CI, *p*	GD	OR, 95 % CI, *p*
***miR-196a-2 (rs11614913) C/T***	CC + CT	412 (84.8)	355 (86.2)	—	344 (84.9)	—	67 (83.8)	—
	TT	74 (15.2)	57 (13.8)	0.89	61 (15.1)	0.99	13 (16.2)	1.31
				(0.62-1.30)		(0.68-1.43)		(0.68-2.53)
				0.556		0.946		0.409
	**Dominant model**							
	**Genotypes**	**Controls**	**RA**	**OR, 95 % CI, *p***	**SLE**	**OR, 95 % CI, *p***	**GD**	**OR, 95 % CI, *p***
	CC	182 (37.4)	142 (34.5)	—	155 (38.3)	—	28 (35.0)	—
	CT + TT	304 (62.6)	270 (65.5)	1.14	250 (61.7)	0.97	52 (65.0)	1.10
				(0.87-1.50)		(0.74-1.27)		(0.68-1.82)
				0.354		0.800		0.674

OR: odds ratio; CI: confidence interval; p: p value; RA: rheumatoid arthritis; SLE: systemic lupus erythematosus; GD: Graves’ disease.

**Table 7:** Association analysis between the *miR-499 rs3746444A/G* polymorphism in RA, SLE and GD patients under different genetic models

Recessive model								
SNP ID	Genotypes	Controls	AR	OR, 95 % CI, *p*	SLE	OR, 95 % CI, *p*	GD	OR, 95 % CI, *p*
***miR-499 (rs3746444) A/G***	AA – AG	485 (99.8)	409 (99.3)	—	407 (100)	—	81 (100)	—
	GG	1 (0.2)	3 (0.7)	3.55	0 (0.0)	—	0 (0.0)	—
				(0.36-34.3)		—		—
				0.24				
	**Dominant model**							
	**Genotypes**	**Controls**	**AR**	**OR, 95 % CI, *P***	**SLE**	**OR, 95 % CI, *P***	**GD**	**OR, 95 % CI, *p***
	AA	425 (87.5)	352 (85.4)	—	336 (82.5)	—	69 (85.2)	—
	AG - GG	61 (12.5)	60 (14.6)	1.18	71 (17.5)	1.47	12 (14.8)	1.21
				(0.81-1.74)		(1.02-2.13)		(0.62-2.36)
				0.379		**0.040**		0.573

OR: odds ratio; CI: confidence interval; p: p value; RA: rheumatoid arthritis; SLE: systemic lupus erythematosus; GD: Graves’ disease.

Significant P-values are reported in bold type.

**Table 8:** Association analysis of polymorphisms in *pre-miR-146a, pre-miR-196a-2* and *pre-miR-499* genes in patients with SLE; no nephritis vs nephritis

Gene SNP	No nephritis	Lupus nephritis	OR	95% CI	*p*
*miR-146a*	n=112 (%)	n=90 (%)			
rs2910164					
Genotype					
GG	41 (36.6)	38 (42.3)	—	—	—
GC	47 (42.0)	40 (44.4)	0.92	0.50-1.69	0.784
CC	24 (21.4)	12 (13.3)	0.54	0.24-1.23	0.139
Allele					
G	129 (57.6)	116 (64.4)	—	—	—
C	95 (42.4)	64 (35.6)	0.75	0.50-1.12	0.161
*miR-196a-2*	n=112 (%)	n=90 (%)			
rs11614913					
Genotype					
CC	46 (41.1)	38 (42.3)	—	—	—
CT	44 (39.3)	40 (44.4)	1.10	0.60-2.02	0.757
TT	22 (19.6)	12 (13.3)	0.66	0.29-1.51	0.322
Allele					
C	136 (60.7)	116 (64.4)	—	—	—
T	88 (39.3)	64 (35.6)	0.85	0.57-1.28	O.442
*miR-499*	n=112 (%)	n=90 (%)			
rs3746444					
Genotype					
AA	94 (83.9)	77 (85.6)	—	—	—
AG	18 (16.1)	13 (14.4)	0.88	0.41-1.91	0.750
GG	0 (0.0)	0 (0.0)	1.22	0.02-62.16	1.000
Allele					
A	206 (92.0)	167 (92.8)	—	—	—
G	18 (8.0)	13 (7.2)	0.89	0.42-1.87	0.760

OR: odds ratio; CI: confidence interval; p: p value; SLE: systemic lupus erythematosus.

## DISCUSSION

In this study, we evaluated the *miR-146a* rs2910164G/C, *miR-196a-2* rs11614913C/T, and *miR-499* rs3746444A/G polymorphisms with regards to their potential associations with RA, SLE, and GD susceptibility in a sample of Mexican patients. Our results revealed that the *miR-146a* rs2910164G/C and *miR-499* rs3746444 polymorphisms were associated with susceptibility to SLE.

Several recent studies have also evaluated the *miR-146a* rs2910164G/C, *miR-196a-2* rs11614913C/T, and *miR-499* rs3746444A/G polymorphisms in patients with RA, SLE, and GD [11–13, 16, 43–46]. Zhou et al. [43] found that the GG genotype of *miR-146a* rs2910164G/C was significantly associated with RA among women. In contrast, we found no association between this SNP and RA susceptibility, before or after gender stratification analysis. Our findings are in accordance with other published studies, including previous investigations in Mexican patients with juvenile rheumatoid arthritis [11–13, 47, 48]. The discrepancy between studies may be at least partly explained by differences in ancestry between studied populations.

The *miR-146a* rs2910164G/C SNP has also been evaluated in Chinese and Sweden populations with SLE, and was not found to be associated with SLE susceptibility in either group [44, 45]. Our present study is the first to document an association between *miR-146a* rs2910164G/C and SLE susceptibility (OR 1.7, 95% CI 1.09–2.61, *p* = 0.017). This polymorphism was also previously evaluated in Mexican patients with pediatric SLE, and no association with susceptibility was identified [48]. This discrepancy could be explained by the genetic differences between adult and pediatric SLE patients. For example, investigations of the *PTPN22* R620W and *TNF-α* −308G/A SNPs in adult Mexican patients with SLE have revealed no associations with susceptibility [49, 50], even though both of these SNPs are associated with SLE susceptibility among pediatric SLE patients [51, 52]. Our study is only the second to evaluates the association between *miR-146a* rs2910164G/C and GD, and we found no association, which is in agreement with the results published by Cai et al. [16].

The functional rs2910164C allele affects the processing and maturation of *miR-146a*, decreasing generation of the mature form compared to with the G allele [53]. Moreover, computational tools predict that the C allele causes mispairing within the mature hairpin [53, 54]. The resulting reduction of functional miR-146a could contribute to the pathogenesis of SLE and other AIDs [55, 56]. In mice, *miR-146a* loss causes spontaneous autoimmunity [57]. MiR-146a negatively regulates the type I interferon (IFN) pathway by targeting IRF5, STAT1, IRAK1, TRAF6, and others. A decrease of miR-146a level results in increases of type I IFN and pro-inflammatory cytokines, such as IL-1β and TNF-α, which are involved in the pathogenesis of various AIDs [58, 59].

The *miR-196a-2* rs11614913C/T polymorphism alters the expression of mature miR-196a and binds to target mRNAs, and this SNP is associated with reduced survival time in patients with non-small-cell lung cancer [60]. A recent evaluation of the *miR-196a-2* rs11614913C/T SNP in RA patients from Egypt found no evidence for association [46], and our present results were in accordance with this finding. Our present study is the first to evaluate this SNP in a population with SLE or GD, and we found no association of this SNP with SLE or GD susceptibility in a Mexican population. This polymorphism should be further investigated in other populations with different ancestries to assess its potential role in RA, SLE, and GD susceptibility.

Three prior studies in Iranian and Egyptian populations report that the *miR-499* rs3746444A/G polymorphism is associated with RA susceptibility, severity, and disease activity; however, no such associations were found in a Han Chinese population [11–13, 46]. Our present findings showed that the *miR-499* rs3746444A/G polymorphism was not associated with RA susceptibility, in line with the findings in a Chinese population [11], and in contrast to the results in Iranian and Egyptian populations [12, 13, 46]. This discrepancy may be related to the differing ancestral backgrounds of the studied populations or to the small sample size. On the other hand, this polymorphism was recently found to be associated with GD susceptibility in Chinese population, while our present results showed no association of this SNP with GD. One major limitation of our present study was the small sample size of GD patients (*n* = 81) compared to the GD patient sample size in the study by Cai et al. (*n* = 701) [16].

Notably, our present study identified an association between the *miR-499* rs3746444A/G polymorphism and SLE susceptibility (AA vs AG; OR 1.5, 95% CI 1.03–2.17, *p* = 0.033). In contrast, a prior study in a Chinese population showed no association between SLE susceptibility and *miR-499* rs3746444A/G [44]. Thus, our present report is the first to document an association between *miR-499* rs3746444A/G and SLE susceptibility. This polymorphism was also investigated in Mexican patients with pediatric SLE, with no detected association. As discussed above, this discrepancy may be explained by genetic background differences between pediatric and adult SLE patients. LN is a major cause of morbidity and mortality in SLE [61]; thus, we evaluated whether the *miR-146a* rs2910164G/C, *miR-196a-2* rs11614913C/T, and *miR-499* rs3746444A/G polymorphisms were associated with LN. Our data showed no association, indicating that these miR-SNPs likely do not confer LN susceptibility.

The rs3746444A/G polymorphism affects the pre-miR-499 stem region, resulting in a change from an A:U to a G:U pairing and mismatching. This alteration reduces the stability of the pre-miR-499 secondary structure. Additionally, the G allele affects miRNA maturation and binding to target mRNAs, increasing susceptibility to various diseases [62], including several AIDs. Qui F et al. recently demonstrated that relative to the *miR-499* rs3746444 A allele, the G allele influences the expression of several genes related to immunity and cancer [63]. Although the role of miR-499 in the pathogenesis of SLE, RA and GD is not well understood, it has been demonstrated that miR-499 targets IL-17RB, IL6, IL-23a, IL-2RB, IL-2, and IL-18R, suggesting its potential to influence these AIDs [8, 11]. Further studies are needed to understand the role of miR-499 in these AIDs.

Our studies in SLE and RA patients reached statistical power (>80%); however, our findings in GD patients could be biased by the small sample size (low statistical power). The cases and controls in all groups were matched by gender, age, and self-reported ancestry. However, it should be noted that the Mexican-Mestizo population is a heterogeneous ethnic group with a very complex genetic structure [64], and thus our findings could be influenced by population stratification. This phenomenon represents a major limitation of this work. Additionally, insufficient clinical data limited our ability to determine whether the investigated polymorphisms could act as modifiers of disease severity in SLE, RA, or GD.

In conclusion, our present results suggest that the functional *miR-146a* rs2910164G/C, *196a-2* rs11614913C/T, and *miR-499* rs3746444A/G polymorphisms were not associated with RA, GD, or LN in patients from Mexico. Our data further suggest that *miR-196a-2* r211614913C/T was not associated with SLE in this population. Importantly, this study is the first to document associations of the *miR-146a* rs2910164G/C and *miR-499* rs3746444A/G polymorphisms with SLE susceptibility.

## MATERIALS AND METHODS

### Study population

Our study population included unrelated subjects over 18 years of age, with a diagnosis of SLE, RA, or GD, recruited from the rheumatology, endocrinology, and immunology outpatient clinics at the Juarez Hospital of Mexico, the National Institute of Cardiology, and the Regional Hospital General in Yucatan. Patients were excluded if they had received a blood transfusion within the last three months; had a viral infection, such as HIV or hepatitis B or C; or had multiple AIDs (except for RA with Sjörgren syndrome). RA patients were classified following the 2010 criteria of the American College of Rheumatology (ACR) [65], GD cases were diagnosed following the criteria of the American Thyroid Association (ATA) [66], and SLE patients were classified based on the 1997 ACR criteria [67]. Data regarding LN presence were available in 202 SLE cases.

From the Juarez Hospital of Mexico, we recruited healthy unrelated control individuals, of over 18 years of age, with self-reported Mexican-Mestizo ancestry (three generations). Control subjects were excluded if they had a family history of autoimmune or chronic inflammatory disease, including obesity, asthma, food allergy, inflammatory bowel disease, chronic urticarial, and others.

All evaluated cases and controls were matched for gender and ethnicity. All study participants provided written informed consent, and this study was approved by the institutional committees of Ethics, Research, and Biosecurity.

### Genomic DNA extraction

From each participant, we obtained a total of 5 ml EDTA-treated peripheral blood. Human nuclear DNA was isolated from PBMCs using an Invisorb Blood Universal Kit (Stratec Molecular GmbH; Berlin, Germany) following the manufacturer's specifications. DNA concentrations were spectrophotometrically measured based on the default OD 260/280 absorbance algorithm. Then the DNA was diluted to a concentration of 5 ng/μl and stored at −20°C until use.

### Determination of polymorphisms in pre-miRNAs

Genotypes of the *miR-146a* rs2910164G/C, *miR-196a-2* rs11614913C/T, and *miR-499* rs3746444A/G SNPs were determined using TaqMan probes (C__15946974_10 for rs2910164, C__31185852_10 for rs11614913, and C___2142612_30 for rs3746444; Applied Biosystems, Foster City, CA) with a CFX96 Touch™ Real-Time PCR Detection System (Bio-Rad, California, USA) following the manufacturer's instructions. Allelic discrimination plots for the three miR-SNPs were constructed using Bio-Rad CFX Manager software. To evaluate the assay reproducibility, 60% of the samples were genotyped twice for all three polymorphisms, showing 100% reproducibility.

### Statistical analysis

To evaluate the HWE for the three investigated miR-SNPs, we used the chi-square test as implemented in the FINETTI software (http://ihg.gsf.de/cgi-bin/hw/hwa1.pl). For all cases and controls, HWE was independently tested for each miR-SNP: *miR-146a* (rs2910164G/C), *miR-196a-2* (rs11614913C/T), and *miR-499* (rs3746444A/G). EPIDAT 3.1 software (http://www.sergas.es/Saude-publica/Epidat-3-1-descargar-Epidat-3-1-(espanol)?print=1) was used to estimate associations between RA, SLE or GD susceptibility and the alleles and genotypes of the evaluated functional miR-SNPs. These analyses included estimation of the odds ratio (OR), 95% confidence interval (95% CI), and *P* value. *P* values of less than 0.05 were considered statistically significant.
